# Trends in contraceptive prevalence rates in sub-Saharan Africa since the 2012 London Summit on Family Planning: results from repeated cross-sectional surveys

**DOI:** 10.1016/S2214-109X(19)30200-1

**Published:** 2019-05-17

**Authors:** Saifuddin Ahmed, Yoonjoung Choi, Jose G Rimon, Souleymane Alzouma, Peter Gichangi, Georges Guiella, Patrick Kayembe, Simon P Kibira, Fredrick Makumbi, Funmilola OlaOlorun, Elizabeth Omoluabi, Easmon Otupiri, Sani Oumarou, Assefa Seme, Solomon Shiferaw, Philip Anglewicz, Scott Radloff, Amy Tsui

**Affiliations:** a Department of Population, Family and Reproductive Health, Bloomberg School of Public Health, Johns Hopkins University, Baltimore, MD, USA; b Institut Supérieur des Sciences de la Population, University of Ouagadougou, Ouagadougou, Burkina Faso; c International Center for Reproductive Health-Kenya, and Technical University of Mombasa, Mombasa, Kenya; d Center for Research, Evaluation Resources and Development, Ile-Ife, Osun State, Nigeria; e Department of Community Medicine, University of Ibadan, Ibadan, Nigeria; f Department of Population, Family and Reproductive Health, Kwame Nkrumah University of Science and Technology, Kumasi, Ghana; g Department of Epidemiology and Biostatistics, Makerere University School of Public Health, Kampala, Uganda; h Department of Community Health, Makerere University School of Public Health, Kampala, Uganda; i Department of Reproductive Health and Health Service Management, School of Public Health, Addis Ababa University, Addis Ababa, Ethiopia; j School of Public Health, University of Kinshasa, Kinshasa, Democratic Republic of the Congo; k Institut National de la Statistique, Niamey, Niger; l Bill & Melinda Gates Institute for Population and Reproductive Health, Department of Population, Family and Reproductive Health, Bloomberg School of Public Health, Johns Hopkins Universiy, Baltimore, MD, USA

## Abstract

**Background:**

The Family Planning 2020 (FP2020) initiative, launched at the 2012 London Summit on Family Planning, aims to enable 120 million additional women to use modern contraceptive methods by 2020 in the world's 69 poorest countries. It will require almost doubling the pre-2012 annual growth rate of modern contraceptive prevalence rates from an estimated 0·7 to 1·4 percentage points to achieve the goal. We examined the post-Summit trends in modern contraceptive prevalence rates in nine settings in eight sub-Saharan African countries (Burkina Faso; Kinshasa, DR Congo; Ethiopia; Ghana; Kenya; Niamey, Niger; Kaduna, Nigeria; Lagos, Nigeria; and Uganda). These settings represent almost 73% of the population of the 18 initial FP2020 commitment countries in the region.

**Methods:**

We used data from 45 rounds of the Performance Monitoring and Accountability 2020 (PMA2020) surveys, which were all undertaken after 2012, to ascertain the trends in modern contraceptive prevalence rates among all women aged 15–49 years and all similarly aged women who were married or cohabitating. The analyses were done at the national level in five countries (Burkina Faso, Ethiopia, Ghana, Kenya, and Uganda) and in selected high populous regions for three countries (DR Congo, Niger, and Nigeria). We included the following as modern contraceptive methods: oral pills, intrauterine devices, injectables, male and female sterilisations, implants, condom, lactational amenorrhea method, vaginal barrier methods, emergency contraception, and standard days method. We fitted design-based linear and quadratic logistic regression models and estimated the annual rate of changes in modern contraceptive prevalence rates for each country setting from the average marginal effects of the fitted models (expressed in absolute percentage points). Additionally, we did a random-effects meta-analysis to summarise the overall results for the PMA2020 countries.

**Findings:**

The annual rates of changes in modern contraceptive prevalence rates among all women of reproductive age (15–49 years) varied from as low as 0·77 percentage points (95% CI −0·73 to 2·28) in Lagos, Nigeria, to 3·64 percentage points (2·81 to 4·47) in Ghana, according to the quadratic model. The rate of change was also high (>1·4 percentage points) in Burkina Faso, Kinshasa (DR Congo), Kaduna (Nigeria), and Uganda. Although contraceptive use was rising rapidly in Ethiopia during the pre-Summit period, our results suggested that the yearly growth rate stalled recently (0·92 percentage points, 95% CI −0·23 to 2·07) according to the linear model. From the meta-analysis, the overall weighted average annual rate of change in modern contraceptive prevalence rates in all women across all nine settings was 1·92 percentage points (95% CI 1·14 to 2·70). Among married or cohabitating women, the annual rates of change were higher in most settings, and the overall weighted average was 2·25 percentage points (95% CI 1·37–3·13).

**Interpretations:**

Overall, the annual growth rates exceeded the 1·4 percentage points needed to achieve the FP2020 goal of 120 million additional users of modern contraceptives by 2020 in the select study settings. Local programme experiences can be studied for lessons to be shared with other countries aiming to respond to unmet demands for family planning. The findings of this study have implications for the way progress is tracked toward achieving the FP2020 goal.

**Funding:**

The Bill & Melinda Gates Foundation.

## Introduction

Since the launch of organised family planning programmes in less industrialised countries in the late 1940s, contraceptive prevalence has increased substantially worldwide, and 54·8% of women of reproductive age (ie, 15–49 years) were using a contraceptive method by 1990.^1^ Contraceptive use improves women's and children's health in many ways, including reducing maternal mortality risks, and improving child survival through birth spacing and the nutritional status of both mothers and children.^2^, ^3^ Additionally, by enabling a shift in the age structure of a population towards a favourable ratio of working population to dependent children, family planning can accelerate fertility declines and spur on the economic development of nations through demographic dividends.^4^, ^5^ Family planning has emerged as a crucial component of sustainable global development and is essential for achieving environmental sustainability.^6^ Unsurprisingly, the US Centers for Disease Control and Prevention termed family planning as one of the top ten great public health achievements in the past century.^7^

**Research in context**

**Evidence before this study**

We searched PubMed for published studies on contraceptive trends using the terms “modern contraceptive prevalence rates”, “mCPR”, AND “trends”, and limited to studies published after 2012. After 2012, two studies were published on trends in contraceptive use for UN countries and Family Planning (FP) 2020-focused countries. One study examined the changes in contraceptive use globally and nationally between 1990 and 2015, based on data from 194 countries, which were collected between 1950 and 2011. The model-based estimates suggested that the contraceptive prevalence rate increased globally only 8·5 percentage points between 1990 and 2010. A systematic analysis of 68 of the 69 FP2020-focused countries published in 2018 suggested that modern contraceptive prevalence rates have increased substantially in some countries, but overall progress is slower than expected to achieve the FP2020 goal of 120 million additional women using a contraceptive method by the year 2020. Another study examined the subnational trends in modern contraceptive prevalence rates in 29 states and union territories in India, the results of which suggested substantial variability in progress across the states.

**Added value of this study**

Demographic and Health Survey and Performance Monitoring and Accountability 2020 websites publish data on modern contraceptive use. This study used data only from the period after the London Summit on Family Planning in 2012 and examined modern contraceptive prevalence rates trends and annual rates of changes after 2012 in nine settings in eight high priority FP2020-focused countries in sub-Saharan Africa. Many countries or areas have substantially increased the prevalence of modern contraceptive use since the FP2020 initiative. The results were not affected by the historical trends and showed the rate of progress for the most recent period after 2012.

**Implications of all the available evidence**

The annual percentage increases were above the average of 1·4 percentage points needed to achieve the 120 million users by 2020 goal (1·92 percentage points for all women and 2·25 percentage points for married or cohabitating women). However, progress is slow in several countries where the annual rate of change is lower than 1 percentage point, far below the target needed to achieve the FP2020 goal. Political disturbances are also likely to have affected the progress of some countries that had shown accelerated progress before 2012.

The progress of family planning, however, began to stall in the early 1990s, perhaps due to the emerging threat of HIV/AIDS at that time shifting global funding priorities: between 1992 and 2005, the percentage share of donor funding in the area of population sectors decreased from 32·1% to 8%.^8^ The donor financing for family planning was US$ 722·8 million in 1998 and was reduced to $393·5 million in 2006.^9^

The London Summit on Family Planning in 2012–organised by the Bill & Melinda Gates Foundation, the UK Government, and other developmental partners–launched the Family Planning 2020 (FP2020) initiative to revitalise the global family planning agenda. The initiative, termed as the rebirth of family planning,^10^ aims to enable an additional 120 million women in using a contraceptive method by the year 2020 in the 69 poorest countries of the world, which have a gross national income per person of less than $2500 per year. The goal is often termed as 120 by 20.^11^ Modern^12^, ^13^ contraceptive prevalence rates in 2012 in these countries was estimated to be 44·3% (95% uncertainty intervals [UI] 42·4–46·0); in African countries, the rate was almost half at 23·9% (95% UI 22·9–25·0).^14^ In middle African countries, the rate was only 9·1%, and in western African countries 12·1%.

Pre-Summit analyses^15^ based on historical data suggested that modern contraceptive method use among married women of reproductive age was increasing annually at a rate of 0·7 percentage points in the 69 poorest countries; the annual rate of change was slightly higher (0·9 percentage points) in sub-Saharan African countries. It was estimated that the annual rate of change needed to double to at least 1·4 percentage points for the 69 poorest countries to achieve the FP2020 goal of enabling an additional 120 million women and girls to use contraceptives by 2020.^11^

A recent analysis suggests that the FP2020 goal is highly ambitious and progress has been slower than expected. Only five of the 68 countries analysed had an annual rate of change of more than 1·4 percentage points between 2012 and 2017: Burkina Faso, Kenya, Malawi, Mozambique, and Uganda (the 69th country, Western Sahara, had no data and was not included in this analysis). Although some countries in sub-Saharan Africa had improved modern contraceptive method use substantially among married women, such as Mozambique and Kenya with annual growth rates of more than 3 percentage points and 2 percentage points, respectively, the overall growth rate was low. The modern contraceptive prevalence rate increased from 23·9% to 28·5% between 2012 and 2017, at less than 1 percentage point annually.^14^ However, this study was based on a few datapoints only, and of the 40 African countries analysed, 29 had none or only one datapoint available for the period after 2012. Moreover, the study results were limited to contraceptive-use analysis among married women only. There is substantial interest in examining the trends of contraceptive use among all women, irrespective of their marital status, since the risk of unwanted pregnancies is high among unmarried sexually active women.

Therefore, we used data from 45 rounds of the Performance and Accountability 2020 (PMA2020) surveys, all undertaken after 2012, to examine the trends in modern contraceptive prevalence rates among all women and married or cohabiting women aged 15–49 years. These data were for women in nine settings in eight countries in the sub-Saharan Africa region, where contraceptive prevalence rates are lowest in all global regions and where governments made official commitment to contribute toward achieving the FP2020 goals during the first year of the initiative. The aim of our study was to examine how annual modern contraceptive prevalence rates have changed in these selected sub-Saharan African settings where data have been collected on a more frequent basis, and how these compare with the minimal 1·4 percentage points annual rate of change necessary for achieving the FP2020 goal.

## Methods

### Study design and data sources

The Demographic and Health Surveys (DHS) have served as the major source of data about contraceptive use for low-income and middle-income countries since the early 1980s. In selected countries, UNICEF's Multiple Indicator Cluster surveys and other national surveys (eg, National Health Surveys; Living Standard Measurement Surveys; and Knowledge, Attitude, and Practice surveys) also collect data on contraceptive use. However, these surveys are done every 5 years or irregularly, and are thus less suitable for timely tracking of FP2020 progress. A rapid-turnaround survey project, PMA2020, was launched in 2013 to fill the gap in survey data for periods that might monitor progress toward the FP2020 goals. The surveys have been done in selected countries where the governments have made political and financial commitments to achieve FP2020 goals locally during the early phase of the partnership. PMA2020 covers the following 11 countries nationally or subnationally to date: Burkina Faso, Côte d'Ivoire, DR Congo, Ethiopia, Ghana, India, Indonesia, Kenya, Nigeria, Niger, and Uganda. Although contraceptive use varies substantially across these countries, the commitments by the governments and stakeholders and corresponding programmatic efforts have generated demand for frequent data collection and monitoring. Additionally, the selected countries are highly populous among the 69 FP2020 priority countries.

The details of PMA2020 survey design and sampling methods are described elsewhere.^16^ In summary, PMA2020 surveys are implemented at the national level in seven countries (Burkina Faso, Côte d'Ivoire, Ethiopia, Ghana, Indonesia, Kenya, and Uganda), and at the selected subnational level in four countries (Kinshasa in DR Congo, Rajasthan in India, Niamey in Niger, and in eight states in Nigeria). In selected countries, additional regions have been included during later rounds. In DR Congo, PMA2020 launched in one province (Kinshasa) first and then expanded to include Kongo Central. In Nigeria, the survey began in two states (Kaduna and Lagos) and subsequently added six states. In India, the PMA2020 survey has been done in one state, Rajasthan. In Niger, PMA2020 is done in Niamey, the capital, with expansion to the national coverage only in alternate survey rounds.

The PMA2020 surveys are repeated cross-sectional surveys based on a multistage stratified cluster sampling design, and the primary sampling units or enumeration areas are selected by probability proportional-to-size method for which the sample selection probability depends on the size of population. To improve the efficiency of the survey design for estimating changes in contraceptive method use, the first four rounds of the surveys in each country were done in the same enumeration areas. This reduced sampling variance of change, given the increased correlation between the successive surveys, and reduced the costs of interviews. However, households were selected randomly and independently in each survey round. Because of concerns of potential interviewee fatigue and learning effect bias among the respondents who might be selected in multiple survey rounds by random chance, it was decided a priori to refresh the enumeration areas after the 4th round. New enumeration areas were independently selected in Ghana, but in other settings, enumeration areas were selected randomly from the listing of all enumeration areas adjacent to the originally selected one. Selection probabilities were reweighted to adjust for the new enumeration areas' measure of size. PMA2020 sampling is usually stratified by regions and urban–rural areas; households are selected randomly within each enumeration area. In selected countries, the survey design was modified in response to the country's needs. In Kenya, counties were represented as strata and 11 were selected with the probability proportional-to-size method. In regional PMA surveys, which often are metropolitan areas (Niamey, Niger; Kinshasa and Kongo Central, DR Congo; Lagos, Nigeria), urban–rural stratification is ignored (Kongo Central was not included in this analysis because data were not available). In Burkina Faso, only urban–rural strata were used. In each primary sampling unit, approximately 33 to 44 households are selected randomly after doing a household listing through a census. All women aged 15–49 who gave consent, irrespective of their marital or cohabitation status, were interviewed by a trained interviewer. The data were collected by mobile phones and uploaded in real time to cloud servers.

The trends analysis of modern contraceptive method use in this Article is limited to nine settings in eight countries in sub-Saharan Africa, where at least four rounds of surveys were done: Burkina Faso; Kinshasa, DR Congo; Ethiopia; Ghana; Kenya; Niamey, Niger; Lagos and Kaduna, Nigeria; and Uganda. 45 surveys were included in the analysis. There were varying definitions of modern contraceptive methods.^12^, ^13^ We included the following contraceptive methods as modern, according to WHO: oral pills, intrauterine devices, injectables, male and female sterilisations, implants, condom, lactational amenorrhea method, vaginal barrier methods, emergency contraception, and standard days method.^17^

### Statistical analysis

We did a design-based analysis^18^ of trends with logistic regression models and adjusted variance estimates with the Taylor series linearisation method for complex survey design. Because modern contraceptive prevalence rates might change linearly or non-linearly, we specified two logistic regression models, linear and quadratic. In the linear model, the time variable was included as a linear function, and in the quadratic model, an additional square term of the time variable (time^2^) is included.

One advantage of these model specifications is that the annual rate of change in modern contraceptive prevalence rates can be estimated from the average marginal effect^19^ of time. Average marginal effect is derived by differentiating dy/dx, the conditional expected values of modern contraceptive prevalence rates with respect to time. It is common practice to estimate the annual rate of change simply by (modern contraceptive prevalence rates_t=T_ – modern contraceptive prevalence rates_t=0_)/T or by log(modern contraceptive prevalence rates_t=T_/modern contraceptive prevalence rates_t=0_)/T. A disadvantage of this approach is that it does not account for any variability or non-linearity in changes within the observed periods. Our measurement is based instead on the average marginal effect that approximately averages the slopes of the change in modern contraceptive prevalence rates across all datapoints. This is an important methodological advantage, because we analysed four to six rounds of surveys over about 4 years in the study countries.

The average marginal effect is approximately equal to β_1_ coefficient when a model is fitted with a linear probability model without a quadratic term. We fitted a logistic regression model, rather than a linear probability model, because logistic regression provided a better prediction fit for binary data.^20^ Although random-coefficient or latent growth models have emerged as an attractive choice for the analysis of individual-level trajectories, these models are not suitable for the analysis of modern contraceptive prevalence rate trajectories for a country or subnational geography because the results are the trajectories of clusters; the modern contraceptive prevalence rate is an individual level estimate of the proportion of women using a modern contraceptive method, not an estimate of cluster average. Moreover, no covariate was included in the model because contraceptive prevalence rate is a crude estimate, not an adjusted or standardised rate.

Another advantage of our approach was that we did the analysis using individual-level data, not aggregate-level estimated data–a common practice for global estimates, which uses the full sample information and is likely to increase statistical power for an inferential test. Both the linear and quadratic logistic regression analyses were done separately by country or subnational geography. We examined the goodness-of-fit F statistics^21^ to see if the model fits were good. We did a random-effects meta-analysis^22^ to summarise the overall annual rate of change in modern contraceptive prevalence rates (weighted by variance) across the PMA2020 survey countries included in this analysis. For the meta-analysis, we selected the linear regression model results when the quadratic term of the time variable was not statistically significant and the results of the quadratic logistic regression model otherwise. Analyses were done separately for all women and for women who were married or living with a partner.

### Role of the funding source

The funder of the study had no role in study design, data collection, data interpretation or writing of the report. The corresponding author had full access to all the data in the study and had final responsibility for the decision to submit for publication.

## Results

**Figure 1:**
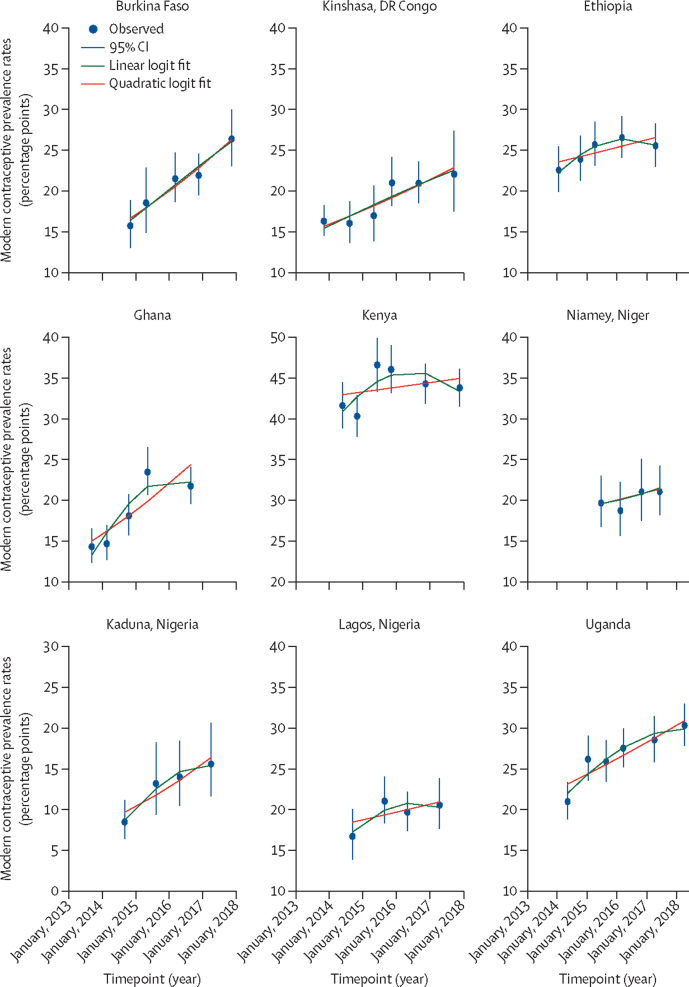
Trends in modern contraceptive prevalence rates for all women aged 15–49 years Data are from Performance Monitoring and Accountability 2020 surveys in nine settings in eight countries in sub-Saharan Africa.

Trends in modern contraceptive prevalence rates for all women aged 15–49 are shown in figure 1 along with the linear and quadratic fitted lines from the logistic regression models. The modern contraceptive prevalence rate trend lines for married or cohabitating women were similar to those for all women (appendix). The fitted lines suggest that modern contraceptive prevalence rates increased in all PMA2020 country settings. Modern contraceptive use at the first round of surveys was lowest in Kaduna, Nigeria and highest in Kenya (table 1).

**Table 1:** Modern contraceptive prevalence rates in eight countries

		**First round**					**Last round**				
		Modern contraceptive prevalence rates (%)		Sample size		Mid-month and year of survey	Modern contraceptive prevalence rates (%)		Sample size		Mid-month and year of survey
		All women	Married or cohabitating women	All women	Married or cohabitating women		All women	Married or cohabitating women	All women	Married or cohabitating women	
Burkina Faso		15·7%	18·0%	2064	1502	November, 2014	26·4%	30·1%	3512	2413	November, 2017
DR Congo											
	Kinshasa	16·2%	18·9%	2129	1073	November, 2013	22·0%	26·7%	2568	1166	September, 2017
Ethiopia		22·5%	32·7%	6468	3670	February, 2014	25·5%	35·2%	7359	4340	May, 2017
Ghana		14·3%	18·7%	3645	2283	September, 2013	21·7%	25·9%	3683	2107	September, 2016
Kenya		41·6%	53·4%	3754	2498	June, 2014	43·7%	59·0%	5876	3404	November, 2017
Niger											
	Niamey	19·7%	28·6%	1336	894	July, 2015	21·1%	32·3%	1330	821	June, 2017
Nigeria											
	Kaduna	8·5%	10·2%	2569	2050	September, 2014	15·6%	18·0%	2838	2206	April, 2017
	Lagos	16·7%	19·7%	764	490	September, 2014	20·5%	23·4%	1509	990	April, 2017
Uganda		21·0%	25·7%	3716	2404	May, 2014	30·3%	36·3%	4225	2674	April, 2018

Data show rates for all women and married or cohabiting women only (percentages are weighted proportions), sample sizes, and survey dates of the first and latest Performance Monitoring and Accountability 2020 rounds in nine settings in eight countries in sub-Saharan Africa.

Improvements in modern contraceptive prevalence rate trends were more pronounced in Burkina Faso, DR Congo (Kinshasa), Nigeria (Kaduna), and Uganda. In Kenya, where contraceptive use was highest among all the PMA2020 countries, modern contraceptive prevalence rates stalled, decreasing from 46·5% in survey round 3 (June to July, 2015) to 43·7% in round 6 (November to December, 2017) among all women. The changes in modern contraceptive prevalence rates in Lagos (Nigeria) were marginal.

**Figure 2:**
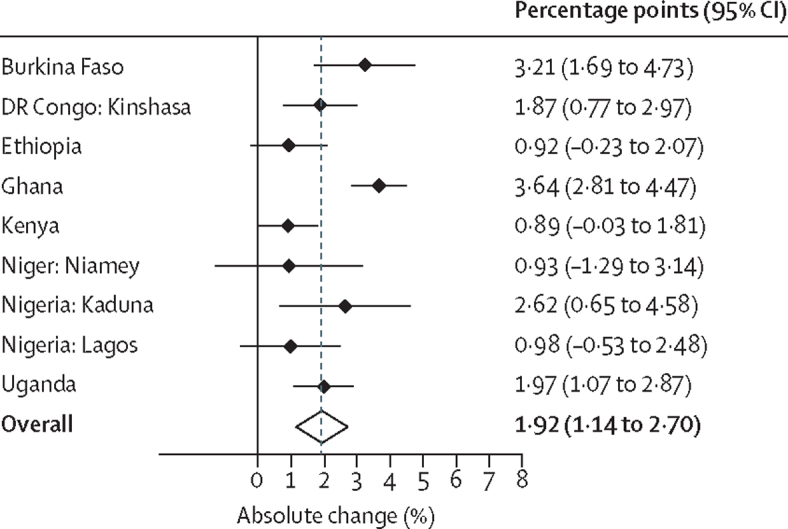
Annual changes in modern contraceptive prevalence rates among all women aged 15-49 years Data are from nine settings in eight Performance Monitoring and Accountability 2020 countries with an overall summary result based on a random-effects meta-analysis.

The goodness-of-fit results suggested that both the linear and quadratic logistic regression model fits were good in all settings (results not shown). The linear model results suggested that the annual rate of change in modern contraceptive prevalence rates among all women was highest in Burkina Faso (table 2). Similarly, according to the linear model a high rate of change occurred in Ghana, Kinshasa (DR Congo), Kaduna (Nigeria), and Uganda. Ethiopia, Kenya, Niamey (Niger), and Lagos (Nigeria) had less than 1 percentage point annual increase in modern contraceptive prevalence rates and the changes were not significant. The annual rates of absolute change results from the quadratic models were similar. The overall modern contraceptive prevalence rate (weighted for the inverse of variance that depends on the sample size of the surveys) increased significantly each year (figure 2). In this analysis, we included the results from the quadratic models where the quadratic term for the time variable was significant but the linear model results were not significant.

**Table 2:** Estimated annual change in modern contraceptive prevalence rates since the 2012 London Summit on Family Planning

		**Linear model (annual rate of change [percentage points {95% CI}])**	**Quadratic model (annual rate of change [percentage points {95% CI}])**
Burkina Faso		3·21 (1·69 to 4·73)	3·17 (1·53 to 4·81)
DR Congo			
	Kinshasa	1·87 (0·77 to 2·97)	1·88 (0·82 to 2·95)
Ethiopia		0·92 (−0·23 to 2·07)	1·13 (0·09 to 2·16)
Ghana		3·06 (2·12 to 3·99)	3·64 (2·81 to 4·47)
Kenya		0·57 (−0·38 to 1·53)	0·89 (−0·03 to 1·81)
Niger			
	Niamey	0·93 (−1·34 to 3·19)	0·93 (−1·26 to 3·14)
Nigeria			
	Kaduna	2·62 (0·63 to 4·61)	2·35 (0·40 to 4·31)
	Lagos	0·98 (−0·53 to 2·48)	0·77 (−0·73 to 2·28)
Uganda		1·97 (1·07 to 2·87)	2·07 (1·22 to 2·93)

Data are average marginal effects for all women aged 15–49 years obtained from the linear and quadratic logistic regression models.

**Figure 3:**
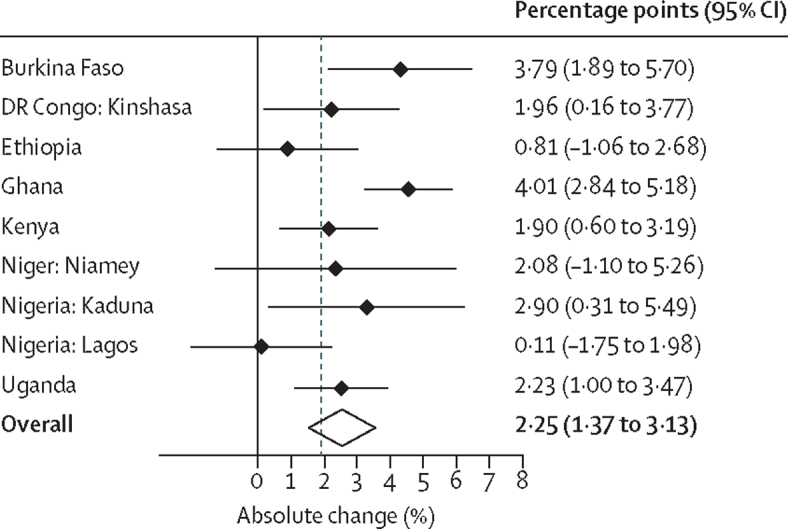
Annual changes in modern contraceptive prevalence rates among married or cohabiting women aged 15–49 years Data are from nine settings in eight Performance Monitoring and Accountability 2020 countries with an overall summary result based on a random-effects meta-analysis.

Except for Ethiopia and Lagos (Nigeria), the annual rate of change in modern contraceptive prevalence rates was more pronounced among married women in all PMA settings (table 3). In Niamey, Niger, the rate of use increased by about 2 percentage points, but this was not significant possibly because of the small sample sizes. In Kenya, the rate of change among married or cohabitating women was almost twice that of all women, suggesting that the increase in contraceptive uptake might not be high among unmarried or non-cohabitating women. The modern contraceptive prevalence rate among married women increased by 4·01 percentage points per year in Ghana, 3·79 percentage points in Burkina Faso, 2·9 percentage points in Kaduna, Nigeria, and about 2 percentage points in Kinshasa, DR Congo; Niamey, Niger; and Kenya. Overall, the modern contraceptive prevalence rate increased by 2·25 percentage points annually among married or cohabitating women (figure 3).

**Table 3:** Estimated annual change in modern contraceptive prevalence rates since the 2012 London Summit on Family Planning

		**Linear model (annual rate of change [percentage points {95% CI}])**	**Quadratic model (annual rate of change [percentage points {95% CI}])**
Burkina Faso		3·79 (1·89 to 5·70)	3·78 (1·71 to 5·86)
DR Congo			
	Kinshasa	1·96 (0·16 to 3·77)	1·95 (0·24 to 3·66)
Ethiopia		0·81 (−1·06 to 2·68)	1·09 (−0·63 to 2·80)
Ghana		3·33 (2·08 to 4·58)	4·01 (2·84 to 5·18)
Kenya		1·34 (0·09 to 2·58)	1·90 (0·60 to 3·19)
Niger			
	Niamey	2·08 (−1·17 to 5·33)	2·09 (−1·10 to 5·26)
Nigeria			
	Kaduna	2·90 (0·31 to 5·49)	2·67 (0·09 to 5·25)
	Lagos	0·43 (−1·52 to 2·38)	0·11 (−1·75 to 1·96)
Uganda		2·23 (1·00 to 3·47)	2·32 (1·15 to 3·49)

Data are average marginal effects for married or cohabitating women aged 15–49 years obtained from the linear and quadratic logistic regression models.

## Discussion

In the nine settings of eight high-priority sub-Saharan countries, PMA2020 data showed that the annual rates of change in modern contraceptive prevalence rates among women of reproductive age (15–49 years) since 2013 have varied substantially––from as low as 0·77 to 3·17 percentage points. Six PMA2020 settings, however, had annual growth rates higher than the 1·4 percentage point change in rates needed to achieve the FP2020 goal of 120 million additional users by year 2020––with four even exceeding 2 percentage points per year, in Burkina Faso, Ghana, Kaduna (Nigeria), and Uganda. Based on weighted averages, the overall absolute annual increase in modern contraceptive prevalence rates in all settings was estimated at 1·92 percentage points for all women and 2·25 percentage points for married or cohabitating women, both higher than the FP2020 1·4 percentage point target. A few countries or subnational regions, however, had slower rates of change, less than 1·4 percentage points per year but higher than the 0·7 percentage points value at the start of FP2020.

Many sub-Saharan African countries have seen the national pace of their modern contraceptive use accelerate. The availability of annual PMA2020 survey data enables the estimation of annual rates of change in modern contraceptive prevalence rates, detecting trends and comparing them against the FP2020 1·4 percentage points goal. Possible explanations for the high rates of growth in modern contraceptive prevalence rates include the introduction of contraceptive implants in the sub-Saharan region and a rise in national commitment to family planning investments. Among the nine settings, the proportion of users of contraceptives relying on implants in the latest PMA2020 survey round ranged from 12·4% in Lagos (Nigeria) to 50·3% in Burkina Faso (data not shown). The annual rate of change in implant use among contraceptives varied from 1·7 percentage points in Burkina Faso to more than 7·0 percentage points in Kaduna (Nigeria) and Kinshasa (DR Congo). The low discontinuation rate for implants over several years of effective use stabilised the modern contraceptive prevalence rate and new adopters each year will raise it.^23^ High discontinuation of injectables and other short-acting methods, by contrast, can reduce the modern contraceptive prevalence rate. In turn, national political commitment to family planning in countries such as Ethiopia, Burkina Faso, DR Congo, and Kenya by governments and non-governmental organisations has increased resource allocations for contraceptive security and delivery. Thus, beyond the growing popularity of implants, proactive local programmes have improved provider training, commodity supply and distribution, and family planning content in mass media, comprising probable reasons behind the robust increases in modern contraceptive prevalence rates.

A primary contribution to this research was our use of recent PMA data since 2012. By contrast, only one round of the DHS data was available after 2012 in five of these countries: DR Congo (2013–14), Ethiopia (2016), Ghana (2014), Niger (2012), and Nigeria (2013). Additionally, Multiple Indicator Cluster surveys (UNICEF) data are available only for Nigeria, which were done in 2016–17. Concerns regarding the few data available initiated the launch of the annual PMA2020 surveys in 2013, making it possible to empirically ascertain the post-2012 Summit changes in modern contraceptive prevalence rates. The data from 45 PMA2020 survey rounds were used in this analysis, with all countries having four to six rounds of surveys.

Our study had some limitations, including that it was based on data from only eight countries in sub-Saharan Africa, which do not fully represent the 69 FP2020 countries. Moreover, these countries pledged to the FP2020 goal and were likely to have committed resources to improve contraceptive use. The progress in non-pledging countries might not be as high as in these eight. Also, all survey data were subject to sampling and non-sampling errors, and some of the temporal variations in modern contraceptive prevalence rates might have been due to noise in the data. Global model-based estimates suggest that modern contraceptive prevalence rates have increased in Kenya about 12·7 percentage points between 2012 and 2017.^14^ However, our analysis showed that the progress in use of modern contraceptives has slowed in Kenya, with modern contraceptive prevalence rates increasing less than 1 percentage point annually. The PMA2020 surveys at health facilities and contraceptive outlets suggest that many facilities are facing stock-outs of contraceptive methods (data not shown). The transition to decentralisation and political activities and strikes by health workers in parts of Kenya, along with growing contraceptive demands, might have affected the supply chain of contraceptive method distribution and procurement.

During the pre-Summit period, modern contraceptive prevalence rates were improving rapidly in Ethiopia, which is the second largest populous country in sub-Saharan Africa: the annual rate of change was 1·4% between 2000 and 2005 and 2·3% between 2005 and 2011 among married women, according to Ethiopia DHS data.^24^, ^25^, ^26^ Our analysis suggested that the annual rate of change in modern contraceptive prevalence rates was only 0·92 percentage points among all women and 0·81 percentage points among married and cohabiting women in the period after the 2012 Summit. The model-based estimate from the UN study^14^ also showed slow progress in changes in modern contraceptive prevalence rates in Ethiopia (6·9 percentage points between 2012 and 2017, 95% CI −0·7 to 14·3). The country has declared states of emergency a few times following political unrest (eg, in 2016 for 10 months, reinstated in 2018), which might have affected family planning and other health and social welfare programmes and services.

Based on annual PMA2020 surveys, our analyses revealed robust upward trends in modern contraceptive use among all reproductive-aged women since 2013, at rates high enough to significantly contribute toward reaching the FP2020 goal. These findings have implications regarding the ways that progress toward the FP2020 goal is being tracked. Further investigation could reveal that progress might have been underestimated and that the robust upward trends might not have been factored in tracking progress. The nine settings invite further examination as to why contraceptive use has risen quickly and the relative effects of demand and supply factors. In particular, the role of newly expanded access to the contraceptive implant, which minimises discontinuation, the mobilisation of community health workers to extend services in rural areas, the deliberate expansion of post-partum programmes in several countries, and the rise of contraceptive social marketing programmes are potential supply side interventions that warrant study in their alignment with trends in contraceptive use.^23^ At present, the ability to empirically measure the satisfaction of growing contraceptive demands serves as a positive signal to the global community that has renewed its commitment to family planning.
