# Supplementary material for: Trends in contraceptive prevalence rates in sub-Saharan Africa since the 2012 London Summit on Family Planning: results from repeated cross-sectional surveys
Source: Lancet Glob Health. 2019 May 17;7(7):e904–11. doi: 10.1016/S2214-109X(19)30200-1 (PMC6560024; doi:10.1016/S2214-109X(19)30200-1)
Supplement: Supplementary appendix [file mmc1.pdf]

THE LANCET
Global Health

Supplementary appendix

This appendix formed part of the original submission and has been peer reviewed. We post it as supplied by the authors.

Supplement to: Ahmed S, Choi Y, Rimon JG, et al. Trends in contraceptive prevalence rates in sub-Saharan Africa since the 2012 London Summit on Family Planning: results from repeated cross-sectional surveys. *Lancet Glob Health* 2019; published online May 17. http://dx.doi.org/10.1016/S2214-109X(19)30200-1.

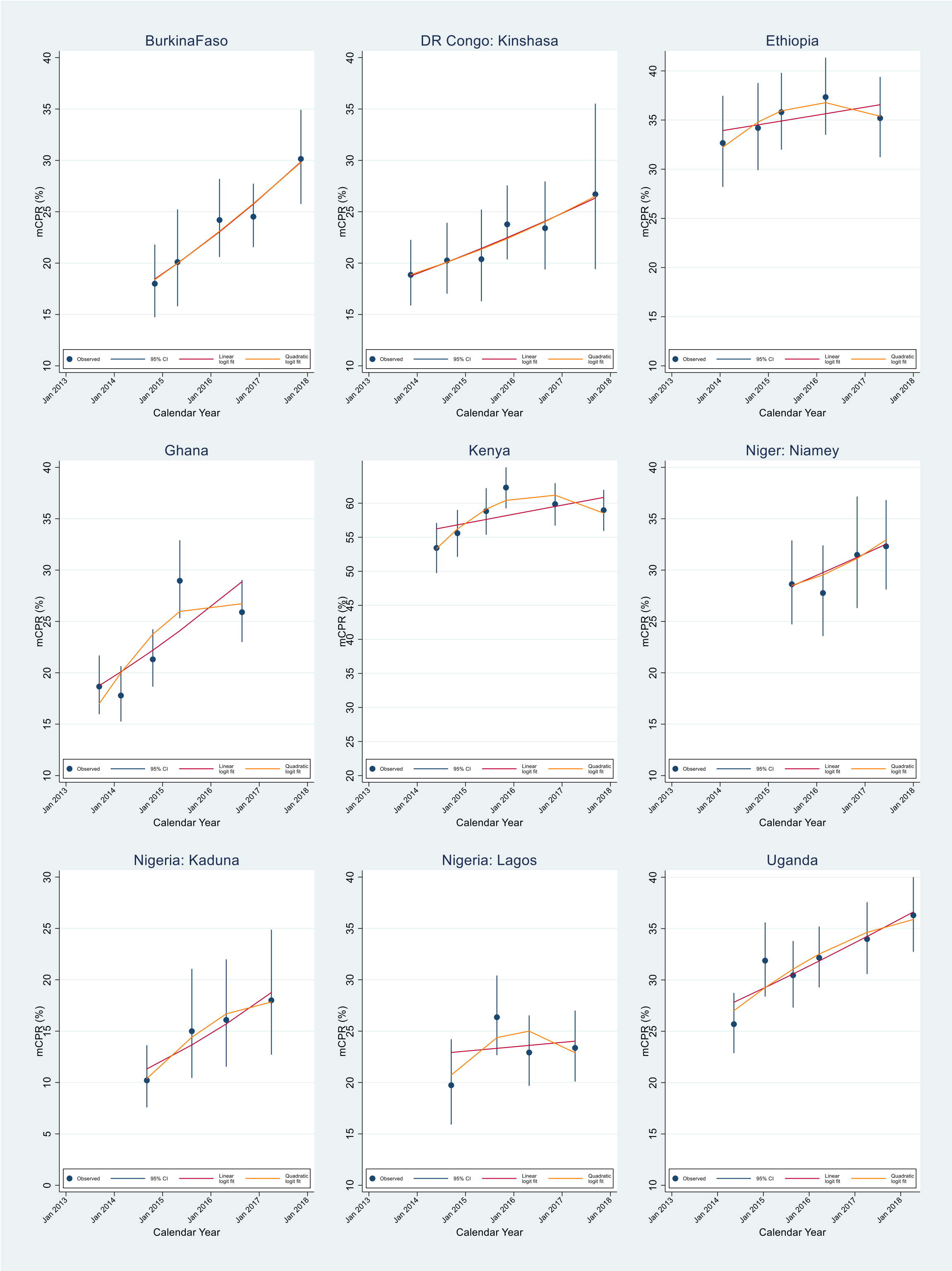

Webappendix: Trends of modern contraceptive prevalence rates among married and cohabitating women of reproductive age 15-49 in 9 settings of 8 PMA2020 countries
